# Supplementary material for: Heterogeneity of human bone marrow and blood natural killer cells defined by single-cell transcriptome
Source: Nat Commun. 2019 Sep 2;10:3931. doi: 10.1038/s41467-019-11947-7 (PMC6718415; doi:10.1038/s41467-019-11947-7)
Supplement: Supplementary file 1 — Supplementary Information [file 41467_2019_11947_MOESM1_ESM.pdf]

Supplementary Information

Heterogeneity of Human Bone Marrow and Blood Natural Killer Cells Defined by Single-cell Transcriptome

Chao Yang[1,2], Jason R Siebert[1,2], Robert Burns[3], Zachary J Gerbec[1,2], Benedetta Bonacci[4], Amy Rymaszewski[5], Mary Rau[6], Matthew J Riese[2,7,8], Sridhar Rao[5,9,10], Karen-Sue Carlson[8,11], John M Routes[5], James W Verbsky[5], Monica S Thakar[1,5], and Subramaniam Malarkannan[1,2,5,8,*]

[1]Laboratory of Molecular Immunology and Immunotherapy, Blood Research Institute, Versiti, Milwaukee, WI;

[2]Departments of Microbiology and Immunology, Medical College of Wisconsin, Milwaukee, WI;

[3]Bioinfomatics Core, Blood Research Institute, Versiti, Milwaukee, WI;

[4]Flow Cytometry Core, Blood Research Institute, Versiti, Milwaukee, WI;

[5]Departments of Pediatrics, Medical College of Wisconsin, Milwaukee, WI;

[6]Departments of Surgery, Medical College of Wisconsin, Milwaukee, WI;

[7]Laboratory of Lymphocyte Biology, Blood Research Institute, Versiti, Milwaukee, WI;

[8]Departments of Medicine, Medical College of Wisconsin, Milwaukee, WI;

[9]Laboratory of Stem Cell Transcriptional Regulation, Blood Research Institute, Versiti, Milwaukee, WI;

[10]Departments of Cell Biology, Neurobiology, and Anatomy, Medical College of Wisconsin, Milwaukee, WI;

[11]Laboratory of Coagulation Biology, Blood Research Institute, Versiti, Milwaukee, WI;

*To whom correspondence should be addressed: S.M. (subra.malar@bcw.edu)

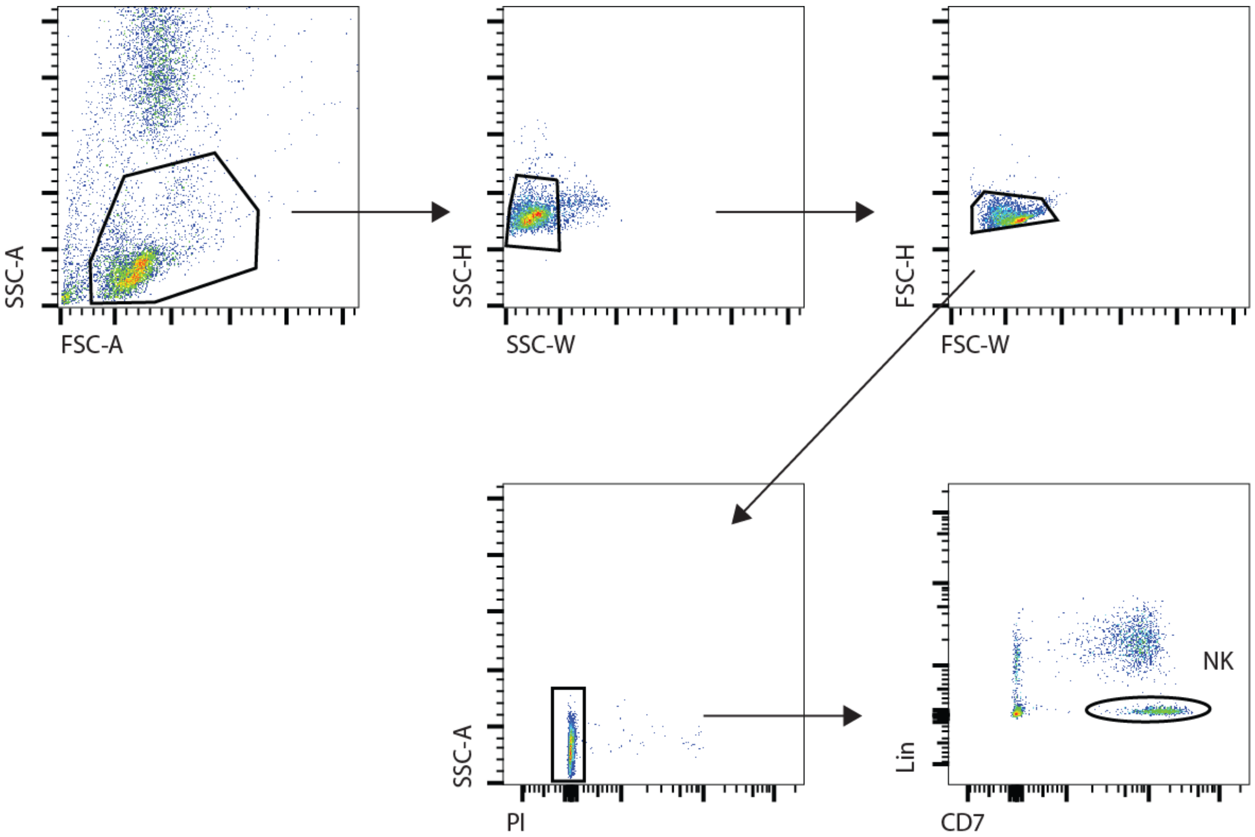

Supplementary Figure 1. Gating strategies used for cell sorting of live human NK cells.

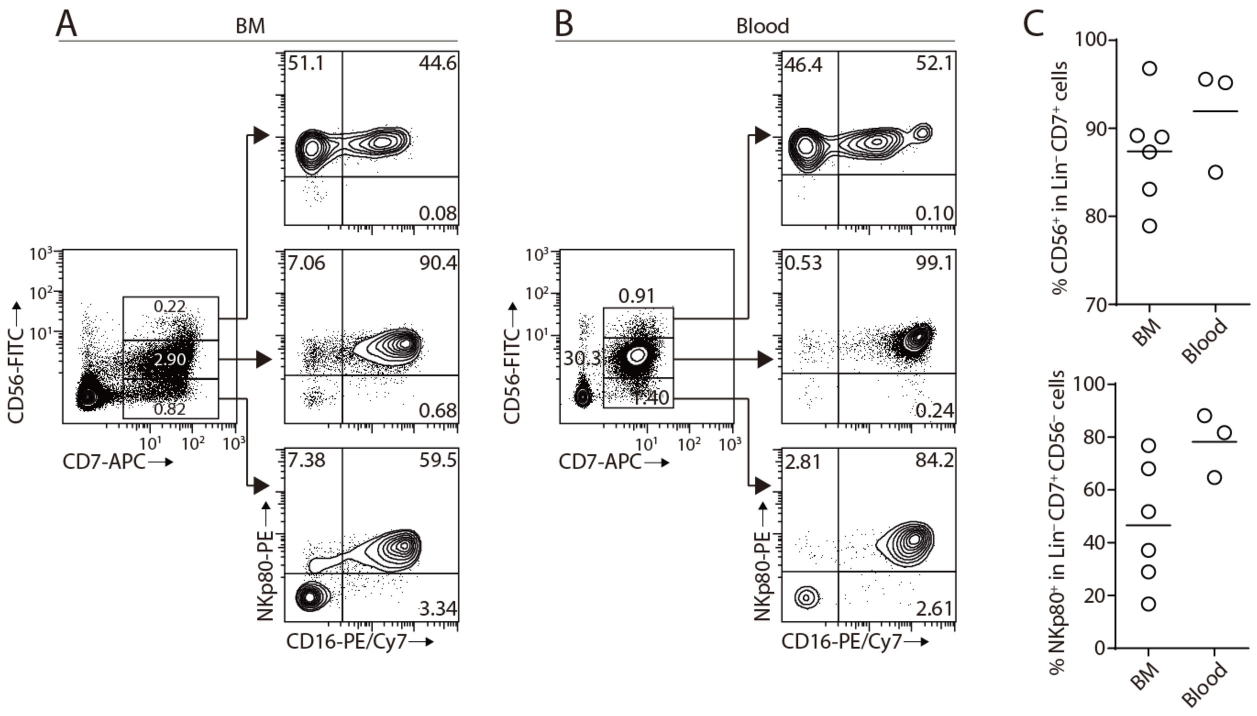

Supplementary Figure 2. Phenotypical analyses of Lin^{-}CD7^{+} cells from BM and blood. (**A**, **B**) Representative flow plots demonstrate the expression of CD56, NKp80, CD16 on Lin^{-}CD7^{+} cells from BM (**A**) or blood (**B**) of healthy donors. (**C**) Percentage CD56^{+} cells within Lin^{-}CD7^{+} population and percentage of NKp80^{+} cells within Lin^{-}CD7^{+}CD56^{-} population were quantified. Source data are provided as a Source Data file.

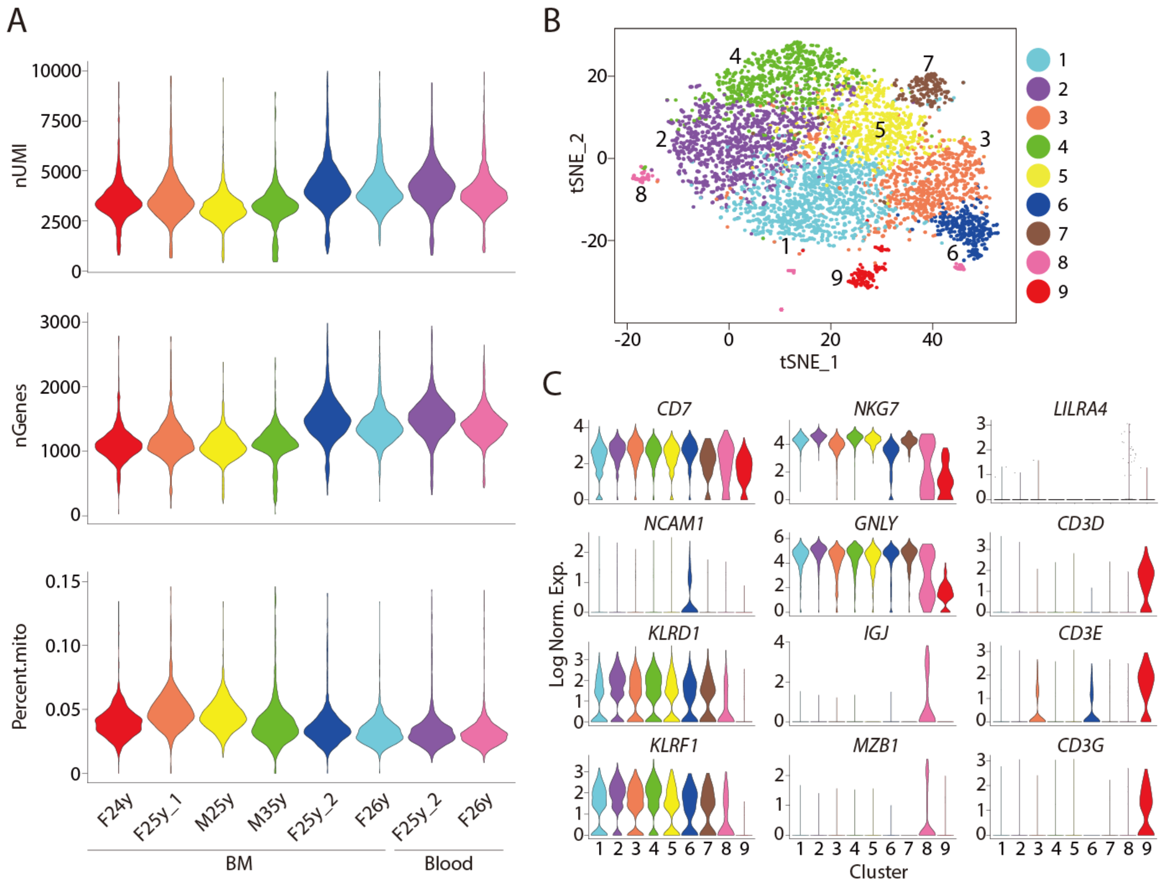

Supplementary Figure 3. Quality control of the scRNA-seq datasets. (**A**) Violin plots demonstrate nUMI, nGene and percentage of mitochondria genes in the transcriptome (percent.mito) of every single cell from either BM or blood of 6 healthy donors without prior filtering. (**B**) Clustering analyses of $Lin^{-}CD7^{+}$ BM cells were illustrated via t-SNE plot. (**C**) The expression of genes defining NK cells, B cells, or T cells in each cluster of $Lin^{-}CD7^{+}$ BM cells were demonstrated via violin plots. The y-axis represents log-normalized expression value.

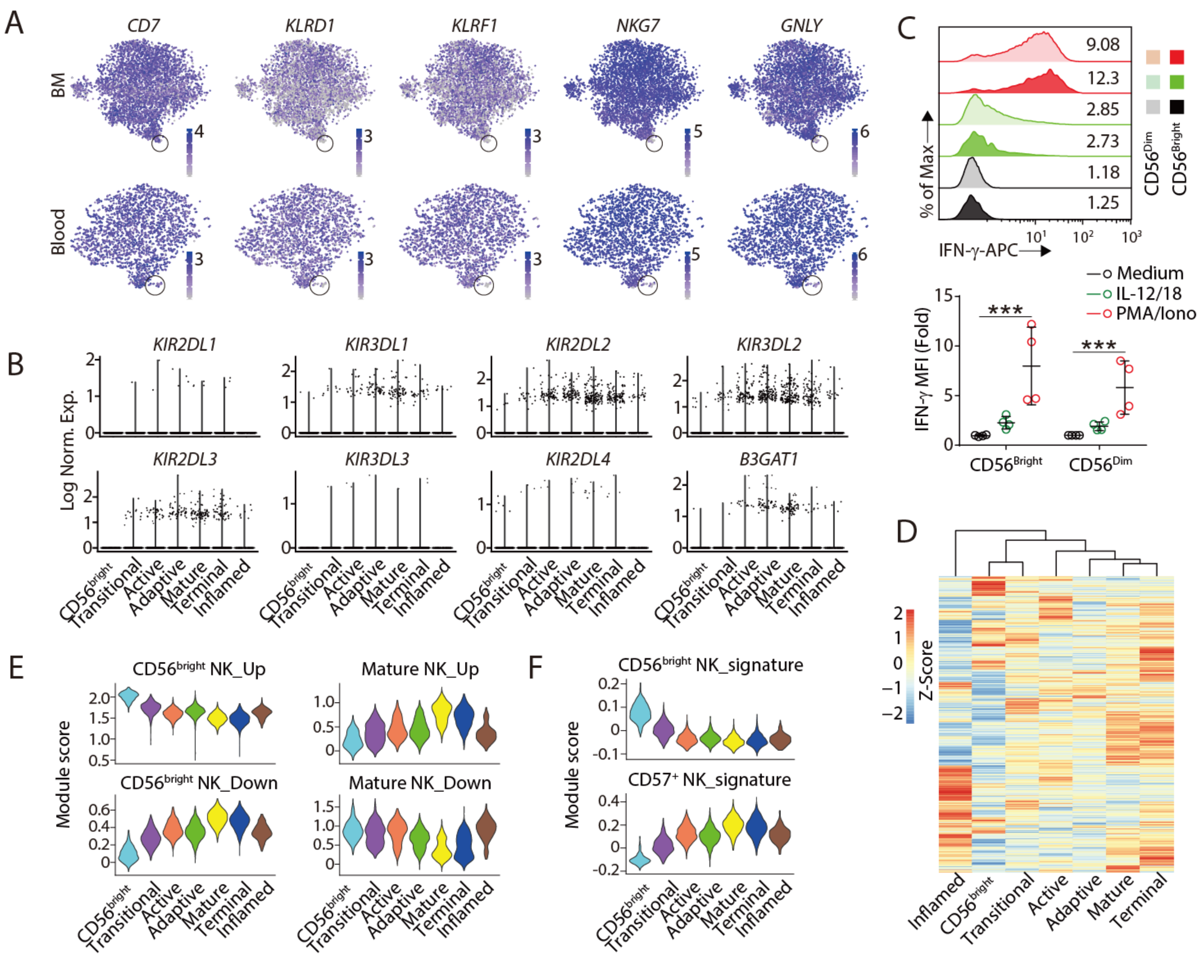

Supplementary Figure 4. Expression of NK-lineage markers and KIRs. (**A**) The expression of markers related to NK-lineage in BM and blood scRNA-seq sample were overlaid to the t-SNE plots. The circle indicated the small fraction of cells that had *CD7*, but not *KLRD1* (CD94), *KLRF1* (NKp80), *NKG7*, or *GNLY* expression compared to the rest of cells. (**B**) The expression of all detectable KIRs and *B3GAT1* (CD57) in the BM sample was plotted as violin plots. The y-axis represents log-normalized expression value. The proportion of cells expressing these genes was not high enough to draw a violin. (**C**) The representative histogram plot on the top demonstrated the production of IFN-γ from CD56^{bright} and CD56^{dim} NK cells stimulated with medium, IL-12/18 (10/10 ng/mL), and PMC/Ionomycin (50/500 ng/mL) for 6 hours. The mean fluorescence intensity (MFI) of each condition was calculated and normalized to the medium-stimulated CD56^{dim} NK cells and summarized on the right. Error bars were shown as standard deviation. n = 4 from three independent experiments. Two-way ANOVA was used for the statistical analysis. *p < 0.05; **p < 0.01; ***p < 0.001. Source data are provided as a Source Data file. (**D**) The transcriptome similarity among clusters of BM sample was evaluated by the Euclidean distance and visualized via heatmap. Each row represents a variable gene among clusters and each column

represents one cluster. (**E**) Module scores were calculated using up-regulated or down-regulated DEGs of ‘$CD56^{bright}$ and Mature NK’ clusters from BM samples and plotted via violin plots. (**F**) Bulk RNA-seq-defined DEGs of $CD56^{bright}$ and $CD56^{dim}CD57^{+}$ NK cells were used to calculate the module score in different clusters of BM samples.

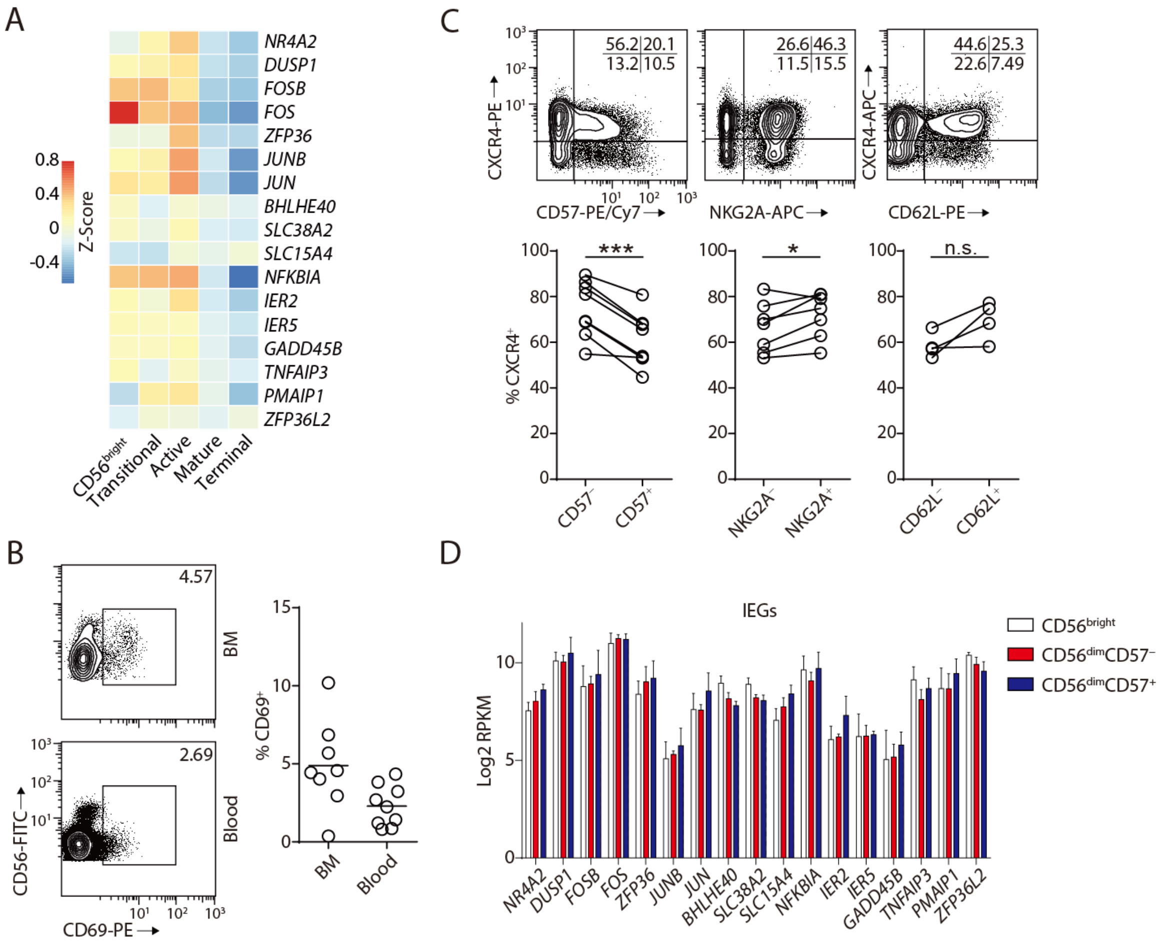

Supplementary Figure 5. Identification of the 'Active NK' cluster featured with expression of IEGs. (**A**) Up-regulated IEGs from the 'Active NK' cluster were plotted using heatmap of the blood sample (**B**) Percentage of CD69$^+$ NK cells (gated on Lin$^-$CD56$^+$ cells) were evaluated via flow cytometry. (**C**) The expression of CXCR4 in CD57$^{+/-}$, CD62L$^{+/-}$, or NKG2A$^{+/-}$ CD56^{dim} NK populations from blood was assessed via flow cytometry (top). Percentage of CXCR4$^+$ cells within each population were quantified (bottom). $n \geq 4$ from four or five independent experiments. Paired student t-test was used for the statistical analysis. *$p < 0.05$; **$p < 0.01$; ***$p < 0.001$. n.s. stands for 'not significant'. (**D**) The transcripts level of IEGs in different NK cell subsets. Source data for (B), (C) and (D) are provided as a Source Data file.

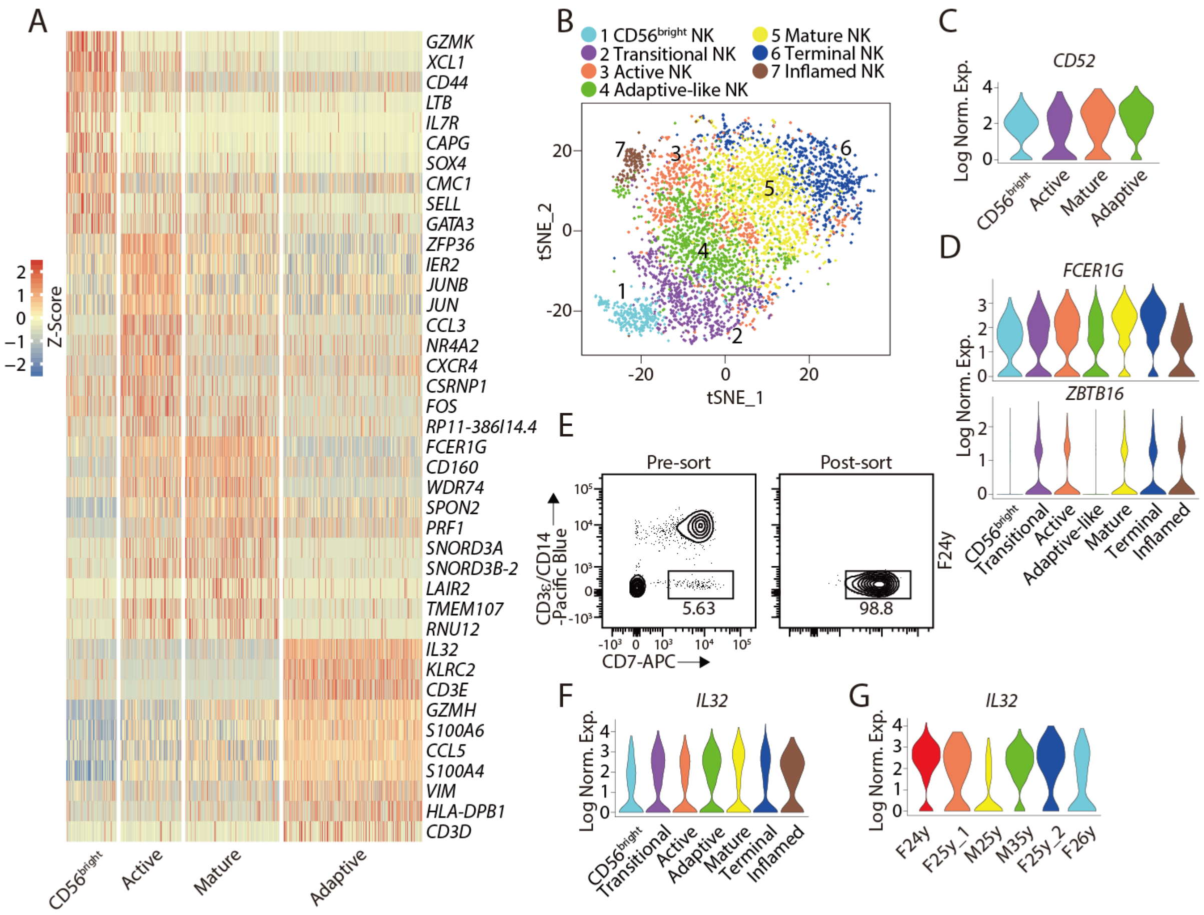

Supplementary Figure 6. Analyses of the 'Adaptive NK' cluster. (**A**) Top 10 up-regulated DEGs (ranked by log fold change) of each cluster from 24-year female sample were plotted using heatmap. (**B**) t-SNE plot demonstrated the 7 clusters from the clustering analyses of the BM sample without the adaptive NK cells from the 24-year old female donor. (**C**) The expression of *CD52* in the four clusters from 24-year female donor sample was shown as a violin plot. The y-axis represents log-normalized expression value. (**D**) Violin plots demonstrated the expression of *FCER1G*, *ZBTB16* in the clusters of the BM sample without the adaptive NK cells from the 24-year old female donor. The y-axis represents log-normalized expression value. (**E**) The purity of the 24-year old female sample was verified after sorting. Percentage of $CD3\varepsilon^{-}CD14^{-}CD7^{+}$ population within $CD19^{-}$ lymphocytes were shown as the flow plots both pre and post sorting. (**F**) Expression of *IL32* in the original BM seven clusters was shown as a violin plot. The y-axis represents log-normalized expression value. (**G**) A violin plot demonstrated the expression of *IL32* in each donor cells from the "Adaptive NK" cluster of the BM sample. The y-axis represents log-normalized expression value.

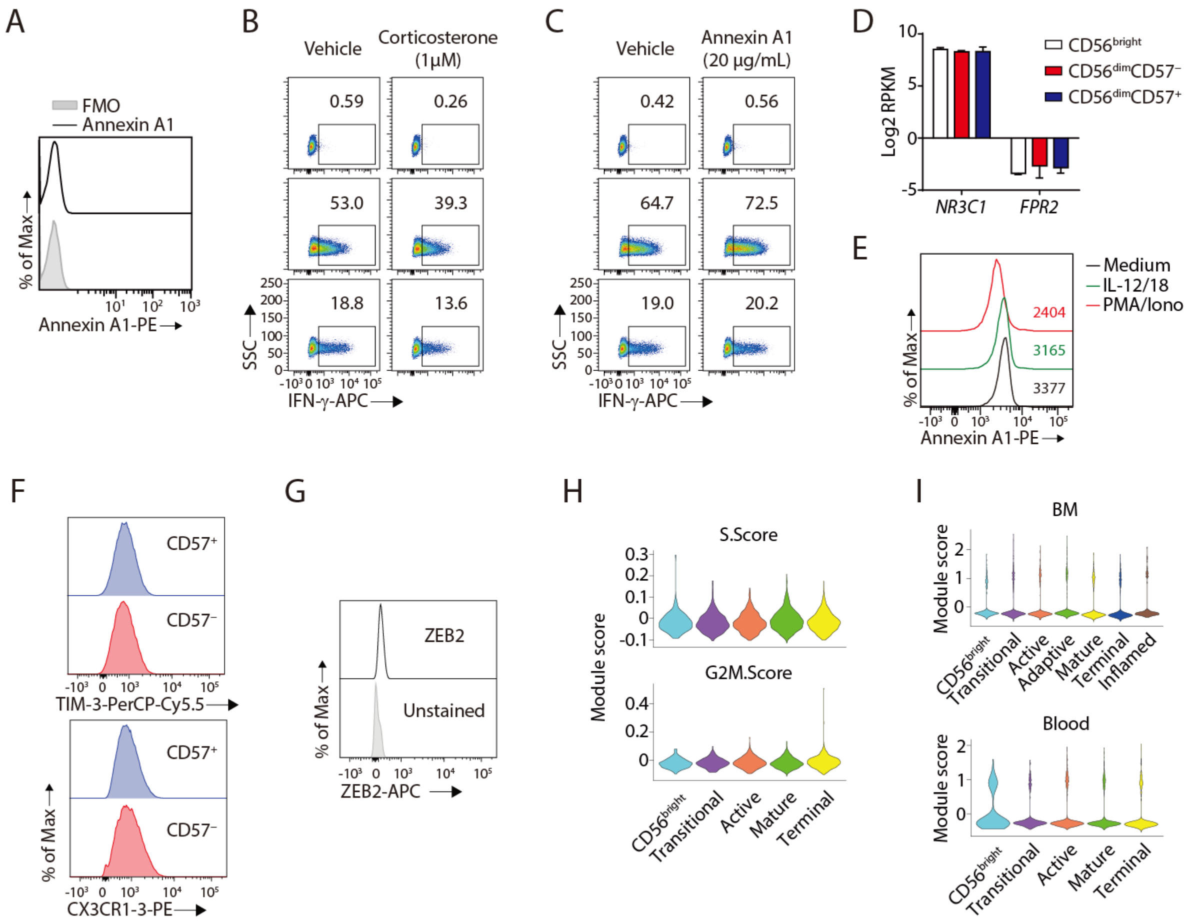

Supplementary Figure 7. Analyses of the 'Mature and Terminal NK' cell cluster. (**A**) Surface staining of Annexin A1on NK cells from blood. FMO stands for fluorescence minus one. (**B, C**) Isolated NK cells from blood were stimulated with medium, IL-12/18 (10/10 ng/mL), and PMA/Ionomycin (50/500 ng/mL) for 6 hours in the presence of 1 µM Corticosterone (**B**) or 20 ug/mL recombinant human Annexin A1 (**C**) and their corresponding vehicle controls. The production of IFN-γ was assessed through intracellular flow analysis. (**D**) The transcripts level of *NR3C1* and *FPR2* in different NK subsets. Error bars were shown as standard deviation. Source data are provided as a Source Data file. (**E**) Isolated NK cells from blood were stimulated with medium, IL-12/18 (10/10 ng/mL), and PMA/Ionomycin (50/500 ng/mL) for 6 hours. The protein level of Annexin A1 was assessed through intracellular flow analysis. (**F**) Gated on $CD56^{dim}$ NK cells, histograms demonstrate the expression of TIM-3 and CX3CR1 on $CD57^{-}$ and $CD57^{+}$ populations. (**G**) Intracellular staining of ZEB-2 on gated $Lin^{-}CD56^{+}$ blood NK cells. (**H**) Cell cycle genes were used to calculate the S.Score and G2M.Score of each cluster in blood sample as shown in violin plots. (**I**) Module scores were calculated using previously defined NK cell quiescence gene set in BM and blood clusters and plotted via violin plots.

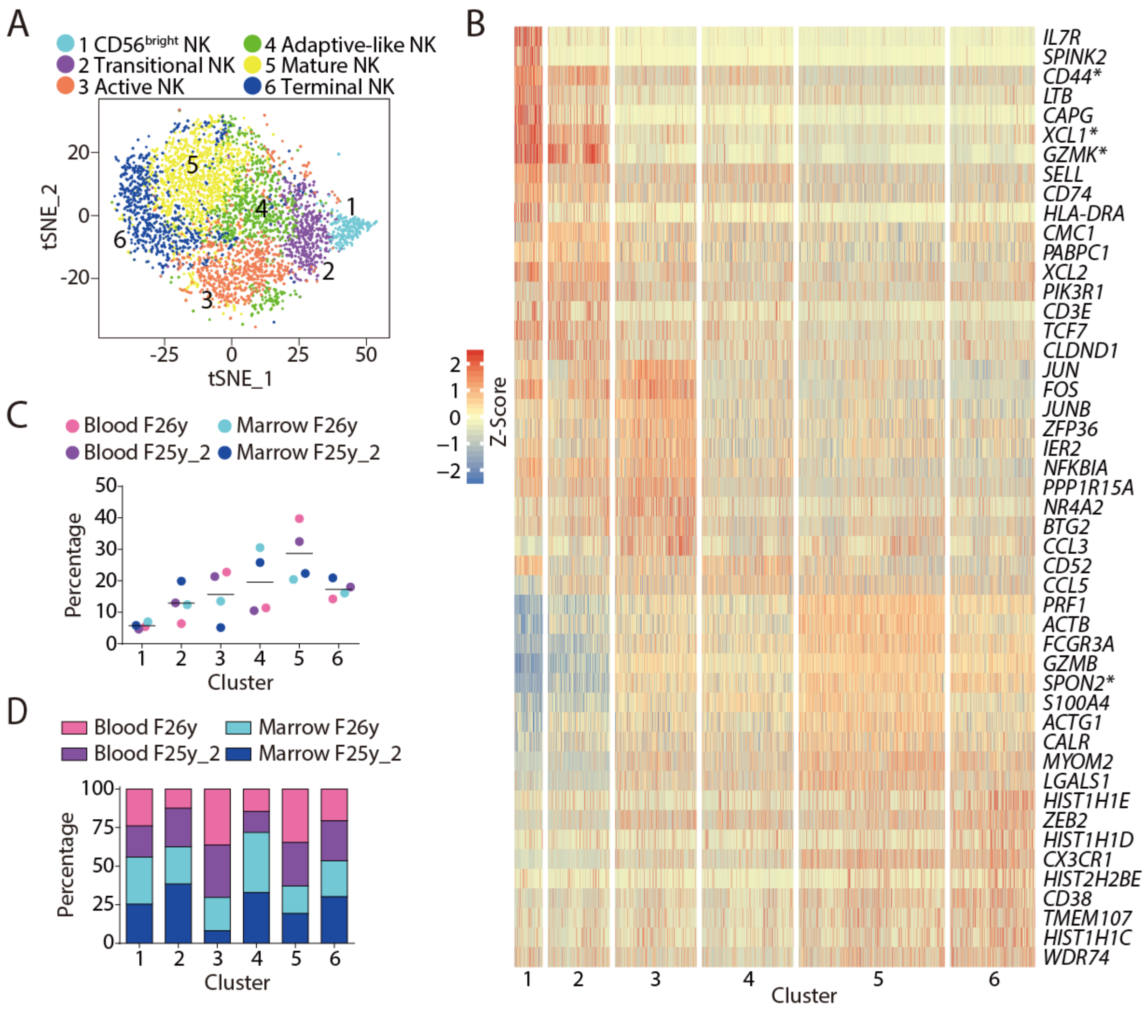

Supplementary Figure 8. Unbiased clustering of human NK cells from BM and blood of same donor. (**A**) Clustering analyses of human NK cells from BM and blood of same donor were performed. Six distinct clusters were numbered, named and demonstrated with t-SNE plot. (**B**) Top 10 up-regulated DEGs (ranked by log fold change) of each cluster were plotted using heatmap. * indicates genes that are DEGs of more than one cluster. (**C**) Composition of the clusters within each donor. (**D**) Composition of the donors within each cluster. The input cell number from each donor is normalized to be equal. Source data for (C) and (D) are provided as a Source Data file.

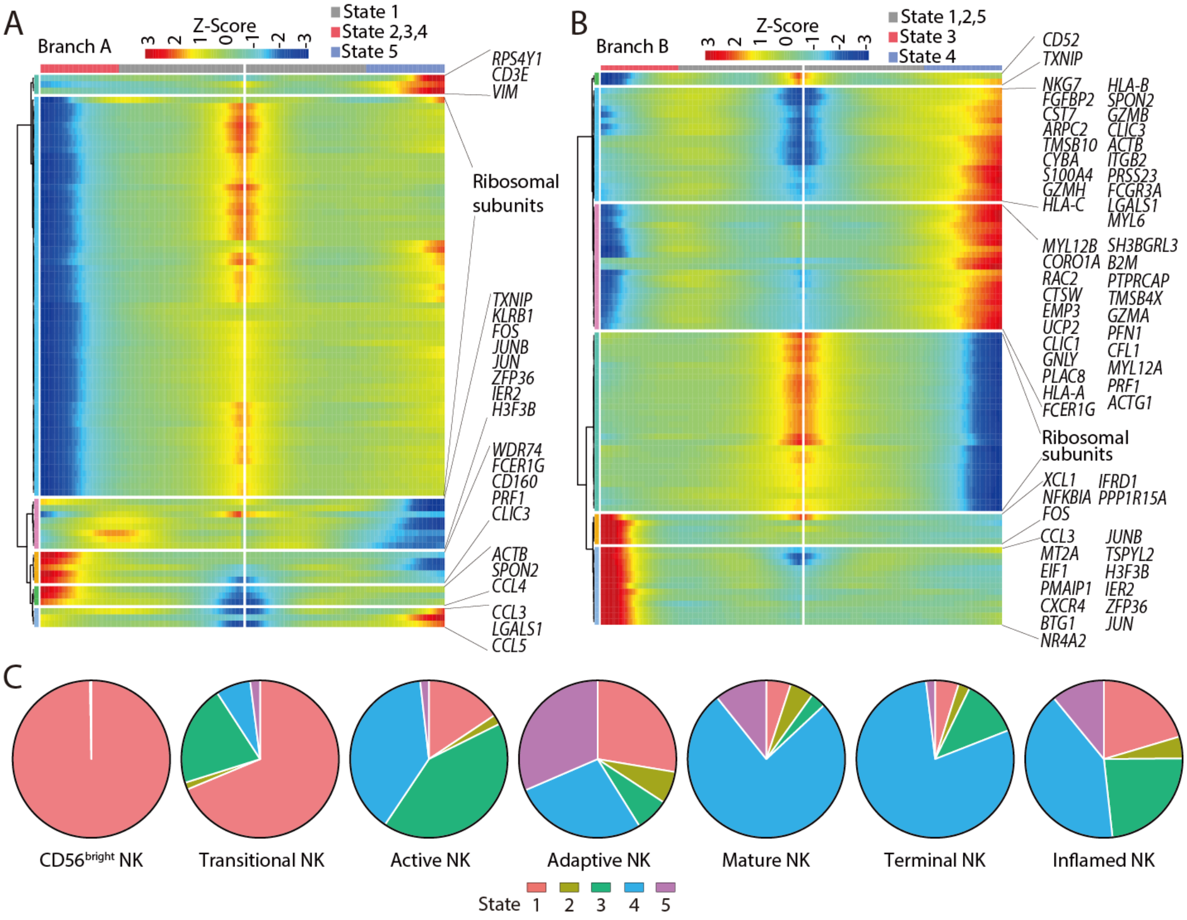

Supplementary Figure 9. Exploration of the branches in the single cell trajectory analysis. (**A**, **B**) The expression of branch point A-dependent (**A**) or branch point B-dependent genes (**B**) were shown in heatmaps. (**C**) Composition of the cell states within each cluster were demonstrated via pie charts. Source data are provided as a Source Data file.

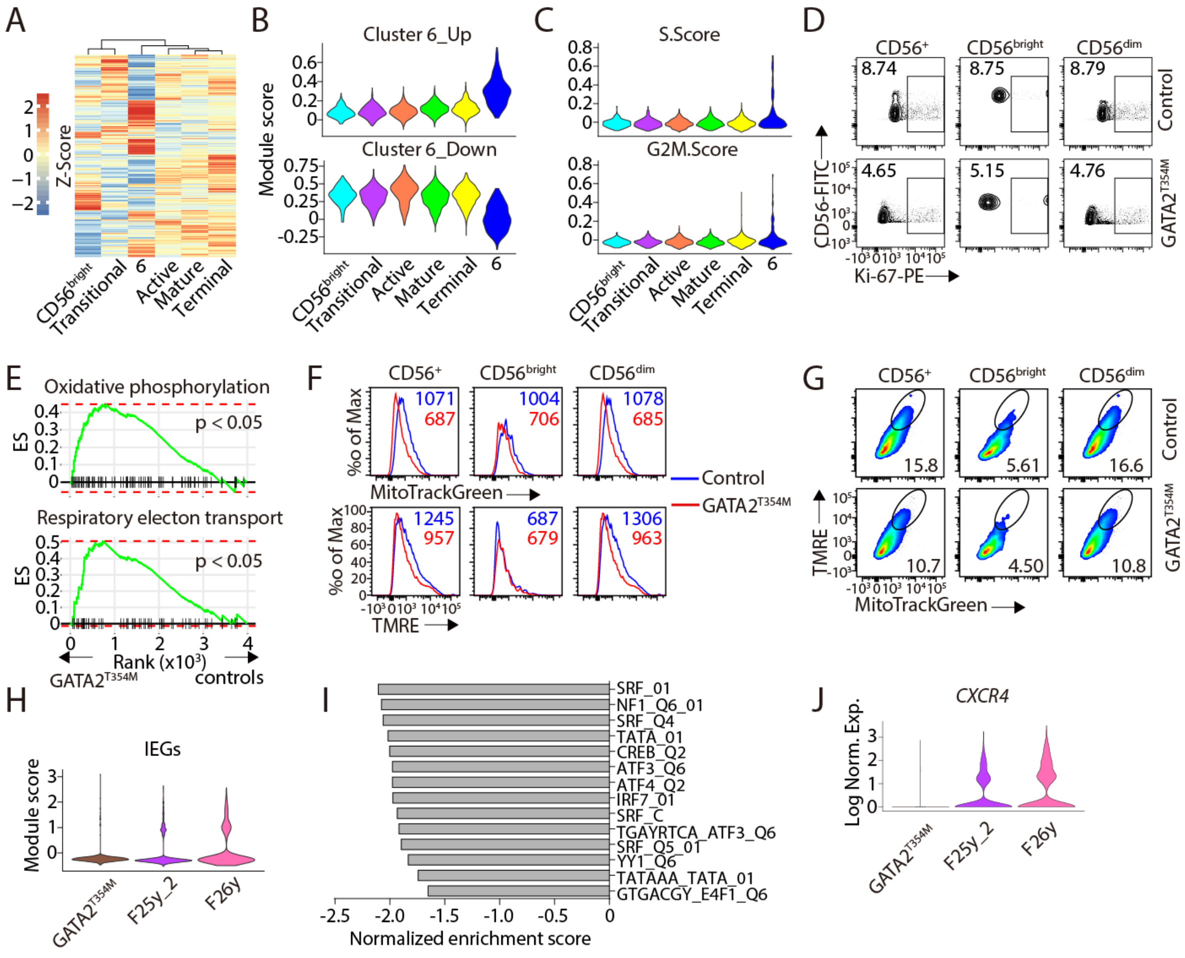

Supplementary Figure 10. Features associated with the GATA2^{T354M} donor NK cells. (**A**) The transcriptome similarity among clusters was compared using the Euclidean distance and demonstrated via heatmap. (**B**) Module score was calculated with up-regulated (top) or down-regulated DEGs (bottom) from the cluster #6 and demonstrated via violin plots. (**C**) The S phase score (top) and G2M phase score (bottom) were calculated and demonstrated via violin plots. (**D**) Proliferating NK cells from the GATA2^{T354M} donor and the healthy control were evaluated via Ki-67 staining. (**E**) GSEA reveals enrichment of oxidative phosphorylation and respiratory electron transport gene sets in the GATA2^{T354M} donor compared to the healthy controls. (**F**) Mitochondrial mass and membrane potential were assessed by MitoTrackGreen and TMRE staining, respectively. (**G**) The polarized mitochondria defined by high mitochondrial mass and membrane potential were gated. (**H**) Module score calculated based on IEGs (as in Fig. 4D) in GATA2^{T354M} donor and the healthy controls. (**I**) All significantly enriched transcription factors and motifs in the GSEA C3 dataset when comparing the GATA2^{T354M} donor with the healthy controls were shown in histogram. (**J**) The expression of *CXCR4* in the GATA2^{T354M} donor and the healthy controls were shown as violin plots. The y-axis represents log-normalized expression value.
