# Supplementary material for: Heterogeneity of human bone marrow and blood natural killer cells defined by single-cell transcriptome
Source: Nat Commun. 2019 Sep 2;10:3931. doi: 10.1038/s41467-019-11947-7 (PMC6718415; doi:10.1038/s41467-019-11947-7)
Supplement: Supplementary file 3 — Reporting Summary [file 41467_2019_11947_MOESM3_ESM.pdf]

nature research

Corresponding author(s): Subramaniam Malarkannan
Last updated by author(s): Jul 15, 2019

Reporting Summary

Nature Research wishes to improve the reproducibility of the work that we publish. This form provides structure for consistency and transparency in reporting. For further information on Nature Research policies, see Authors & Referees and the Editorial Policy Checklist.

Statistics

For all statistical analyses, confirm that the following items are present in the figure legend, table legend, main text, or Methods section.

n/a	Confirmed	
☐	☒	The exact sample size (*n*) for each experimental group/condition, given as a discrete number and unit of measurement
☐	☒	A statement on whether measurements were taken from distinct samples or whether the same sample was measured repeatedly
☐	☒	The statistical test(s) used AND whether they are one- or two-sided<br>*Only common tests should be described solely by name; describe more complex techniques in the Methods section.*
☒	☐	A description of all covariates tested
☒	☐	A description of any assumptions or corrections, such as tests of normality and adjustment for multiple comparisons
☐	☒	A full description of the statistical parameters including central tendency (e.g. means) or other basic estimates (e.g. regression coefficient) AND variation (e.g. standard deviation) or associated estimates of uncertainty (e.g. confidence intervals)
☐	☒	For null hypothesis testing, the test statistic (e.g. *F*, *t*, *r*) with confidence intervals, effect sizes, degrees of freedom and *P* value noted<br>*Give P values as exact values whenever suitable.*
☐	☒	For Bayesian analysis, information on the choice of priors and Markov chain Monte Carlo settings
☒	☐	For hierarchical and complex designs, identification of the appropriate level for tests and full reporting of outcomes
☒	☐	Estimates of effect sizes (e.g. Cohen's *d*, Pearson's *r*), indicating how they were calculated

Our web collection on statistics for biologists contains articles on many of the points above.

Software and code

Policy information about availability of computer code

Data collection	No software was used.
Data analysis	10X Genomics Cell Ranger pipeline (v2.1.1), Seurat package (v2.3.1) and Monocle2 (v2.6.4) in R (v3.4.3 or above)

For manuscripts utilizing custom algorithms or software that are central to the research but not yet described in published literature, software must be made available to editors/reviewers. We strongly encourage code deposition in a community repository (e.g. GitHub). See the Nature Research guidelines for submitting code & software for further information.

Data

Policy information about availability of data

All manuscripts must include a data availability statement. This statement should provide the following information, where applicable:

- Accession codes, unique identifiers, or web links for publicly available datasets
- A list of figures that have associated raw data
- A description of any restrictions on data availability

All the sequencing data is uploaded to NCBI Gene Expression Omnibusequence (GEO). The accession code is GSE130430. The data will be released to public upon manuscript acceptance for publication

Field-specific reporting

Please select the one below that is the best fit for your research. If you are not sure, read the appropriate sections before making your selection.

☒ Life sciences ☐ Behavioural & social sciences ☐ Ecological, evolutionary & environmental sciences

Life sciences study design

All studies must disclose on these points even when the disclosure is negative.

Sample size	For scRNA-sequencing, we analyzed human BM from six healthy donors and blood from two healthy donors. Each sample, we sequenced > 1,000 NK cells. Therefore, we collected RNAseq data from a total of more than 8,000 cells, which is statistically sufficient to cover the variations and to make significant conclusions. For flow cytometry analyses, we included at least 3 individual human samples for each conditions. This falls within the standard practice for analyzing human samples.
Data exclusions	No data were excluded from the study
Replication	This current work largely consists of scRNA-sequencing. Data replication was performed by sequencing > 1,000 NK cells per individual human sample.
Randomization	This is not relevant to the study as there is no group allocation.
Blinding	Blinding was not relevant to the study as there is no group allocation.

Reporting for specific materials, systems and methods

We require information from authors about some types of materials, experimental systems and methods used in many studies. Here, indicate whether each material, system or method listed is relevant to your study. If you are not sure if a list item applies to your research, read the appropriate section before selecting a response.

Materials & experimental systems

n/a	Involved in the study	
☐	☒	Antibodies
☒	☐	Eukaryotic cell lines
☒	☐	Palaeontology
☒	☐	Animals and other organisms
☒	☐	Human research participants
☒	☐	Clinical data

Methods

n/a	Involved in the study	
☒	☐	ChIP-seq
☐	☒	Flow cytometry
☒	☐	MRI-based neuroimaging

Antibodies

Antibodies used	CD3E (UCHT1), CD19 (HIB19), CD14 (HCD14), CD20 (2H7), CD34 (581), CD7 (CD7-6B7), NKp80 (5D12), CD16 (B73.1), CD44 (IM7), CXCR4-biotin (12G5), CD57 (HNK-1), TCRA/B (IP26), TCRG/D (B1), Streptavidin-PE/AF647 from Biolegend (San Diego, CA); CD56 (TULY56), CD69 (FN50), CD62L (Dreg56), Ki-67 (SolA15), IFNgamma (4S.B3), MitoTrackGreen, TMRE are from Thermo-Fisher Scientific (Waltham, MA); NKG2A (REA110), NKG2C (REA205) are from Miltenyi Biotec (Bergisch Gladbach, Germany); Annexin A1 (D5V2T) is from Cell Signaling (Danvers, MA); XCL1 is from R & D (Minneapolis, MN).
Validation	All antibodies are validated by the manufactures.

Flow Cytometry

Plots

Confirm that:

☒ The axis labels state the marker and fluorochrome used (e.g. CD4-FITC).

☒ The axis scales are clearly visible. Include numbers along axes only for bottom left plot of group (a 'group' is an analysis of identical markers).

☒ All plots are contour plots with outliers or pseudocolor plots.

☒ A numerical value for number of cells or percentage (with statistics) is provided.

Methodology

Sample preparation	BM and blood sample were diluted with ice-cold PBS containing 2 mM EDTA and carefully layered over lymphoprep, and then centrifuged at 440 × g for 35 min at 20 °C without brake. After aspirating the upper layer, mononuclear cells at the interphase were carefully transferred and washed once with PBS containing 2 mM EDTA before downstream process.
Instrument	LSR-II (BD Biosciences, San Jose, CA) or MACSQuant Analyzer 10 (Miltenyi Biotec, Bergisch Gladbach, Germany)

Software	FlowJo software (FlowJo LLC, Ashland, OR)
Cell population abundance	We generally recover at least 100,000 cells of the relevant population post-sorting. We conducted post-sorting purity check via flow analyses of the post-sorting fraction. The purity is above 95%.
Gating strategy	A tight lymphocytes gate was used in the FSC/SSC plot. The boundaries between "negtive" and "positive" were determined based on isotype control antibody staining.

☒ Tick this box to confirm that a figure exemplifying the gating strategy is provided in the Supplementary Information.
