# Supplementary material for: Heterogeneity of human bone marrow and blood natural killer cells defined by single-cell transcriptome
Source: Nat Commun. 2019 Sep 2;10:3931. doi: 10.1038/s41467-019-11947-7 (PMC6718415; doi:10.1038/s41467-019-11947-7)
Supplement: Supplementary file 4 — Description of Additional Supplementary Files [file 41467_2019_11947_MOESM4_ESM.pdf]

**Description of Additional Supplementary Files**

File Name: Supplementary Data 1

Description: DEGs of each cluster in the bone marrow sample (p_val_adj < 0.05). Related to Fig. 1

File Name: Supplementary Data 2

Description: DEGs of each cluster in the blood sample (p_val_adj < 0.05). Related to Fig. 2

File Name: Supplementary Data 3

Description: Significantly enriched pathways comparing cluster 1 ($CD56^{bright}$ NK) to the rest of cells in the marrow sample (padj < 0.05). Related to Fig. 3

File Name: Supplementary Data 4

Description: Significantly enriched pathways comparing cluster 1 ($CD56^{bright}$ NK) to the rest of cells in the blood sample (padj < 0.05). Related to Fig. 3

File Name: Supplementary Data 5

Description: Significantly enriched pathways comparing cluster 7 (inflamed NK) to the rest of cells in the marrow sample (padj < 0.05). Related to Fig. 4

File Name: Supplementary Data 6

Description: Significantly enriched pathways comparing cluster 3 (active NK) to the rest of cells in the marrow sample (padj < 0.05). Related to Fig. 4

File Name: Supplementary Data 7

Description: Significantly enriched pathways comparing cluster 3 (active NK) to the rest of cells in the blood sample (padj < 0.05). Related to Fig. 4

File Name: Supplementary Data 8

Description: DEGs of each cluster in the bone marrow sample without adaptive NK cells from 24-year female (p_val_adj < 0.05). Related to Fig. S5

File Name: Supplementary Data 9

Description: Significantly enriched pathways comparing cluster 5 (mature NK) to the rest of cells in the marrow sample (padj < 0.05). Related to Fig. 6

File Name: Supplementary Data 10

Description: Significantly enriched pathways comparing cluster 4 (mature NK) to the rest of cells in the blood sample (padj < 0.05). Related to Fig. 6

File Name: Supplementary Data 11

Description: DEGs comparing all NK cells from the GATA2T354M donor to all NK cells from the healthy control donors (p_val_adj < 0.05, ranked by avg_logFC). Related to Fig. 8
